# Artificial intelligence and machine learning in prehospital emergency care: A scoping review

**DOI:** 10.1016/j.isci.2023.107407

**Published:** 2023-07-17

**Authors:** Marcel Lucas Chee, Mark Leonard Chee, Haotian Huang, Katelyn Mazzochi, Kieran Taylor, Han Wang, Mengling Feng, Andrew Fu Wah Ho, Fahad Javaid Siddiqui, Marcus Eng Hock Ong, Nan Liu

**Affiliations:** 1 Faculty of Medicine, Nursing and Health Sciences, Monash University, Melbourne, VIC, Australia; 2 Faculty of Health and Medical Sciences, University of Adelaide, Adelaide, SA, Australia; 3 Saw Swee Hock School of Public Health, National University of Singapore, Singapore, Singapore; 4 Department of Emergency Medicine, Singapore General Hospital, Singapore, Singapore; 5 Pre-Hospital and Emergency Research Centre, Duke-NUS Medical School, Singapore, Singapore; 6 Centre for Quantitative Medicine, Duke-NUS Medical School, Singapore, Singapore; 7 Institute of Data Science, National University of Singapore, Singapore, Singapore

**Keywords:** Emergency medicine, Preventive medicine, Artificial intelligence applications

## Abstract

Our scoping review provides a comprehensive analysis of the landscape of artificial intelligence (AI) applications in prehospital emergency care (PEC). It contributes to the field by highlighting the most studied AI applications and identifying the most common methodological approaches across 106 included studies. The findings indicate a promising future for AI in PEC, with many unique use cases, such as prognostication, demand prediction, resource optimization, and the Internet of Things continuous monitoring systems. Comparisons with other approaches showed AI outperforming clinicians and non-AI algorithms in most cases. However, most studies were internally validated and retrospective, highlighting the need for rigorous prospective validation of AI applications before implementation in clinical settings. We identified knowledge and methodological gaps using an evidence map, offering a roadmap for future investigators. We also discussed the significance of explainable AI for establishing trust in AI systems among clinicians and facilitating real-world validation of AI models.

## Graphical abstract

**Figure:**
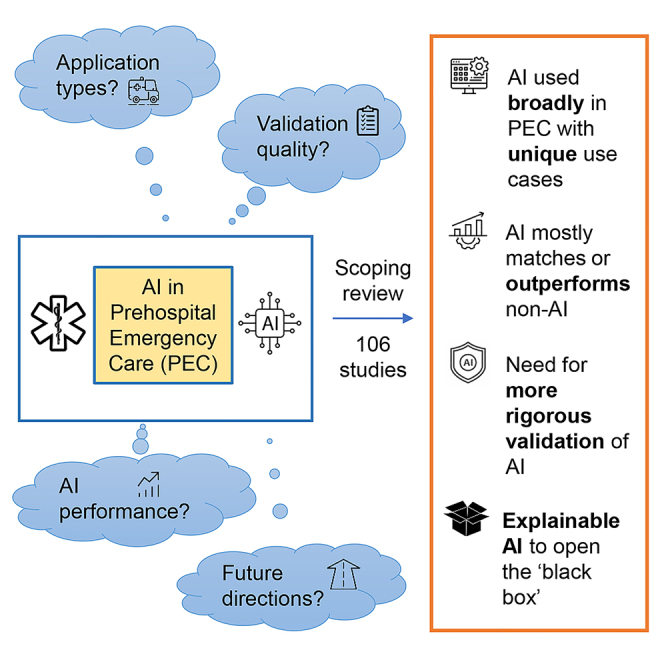

## Introduction

Artificial intelligence (AI) and machine learning (ML) are at the forefront of digital medicine.^1^^,^^2^ They have been extensively applied to various medical domains such as cardiology,^3^ ophthalmology,^4^ emergency medicine,^5^^,^^6^ and many others. As summarized in numerous reviews and discussions on the adoption of AI and ML techniques in healthcare, both structured and unstructured data (e.g., medical images, clinical free texts, time-series physiological signals) benefit from the versatility and flexibility of AI and ML techniques. In addition to healthcare institution-based applications, the intersection of the Internet of Things (IoT) and AI have also attracted interest in the form of wearables and remote continuous health monitoring.^7^

While there have been attempts to summarize the evidence on AI and ML applications in acute care,^5^^,^^6^^,^^8^^,^^9^^,^^10^^,^^11^^,^^12^ little is reported on their use in prehospital emergency care (PEC) settings. Adoption of AI solutions in PEC is hindered by limited resources and the fast-paced nature of PEC workflows. PEC systems are further complicated by the need for coordination and collaboration between multiple disciplines, such as emergency medicine, critical care, disaster management, and transportation networks. Despite growing research into AI and ML in PEC, a systematic review and summary of relevant literature are lacking, making it difficult to understand the current state and future directions for the field.

In this paper, we present a scoping review of six databases (MEDLINE, Embase, Scopus, IEEE Xplore, ACM Digital Library, and Cochrane Central Register of Controlled Trials (CENTRAL)) to summarize the current literature on AI and ML applications in PEC research. The aims of the study are to provide a descriptive analysis of the current literature, and to visualize and identify knowledge and methodological gaps using an evidence map.^13^^,^^14^ The evidence map categorizes studies by both applications and input data, allowing a granular analysis of gaps in the current literature.

## Results

### Studies included

**Figure 1:**
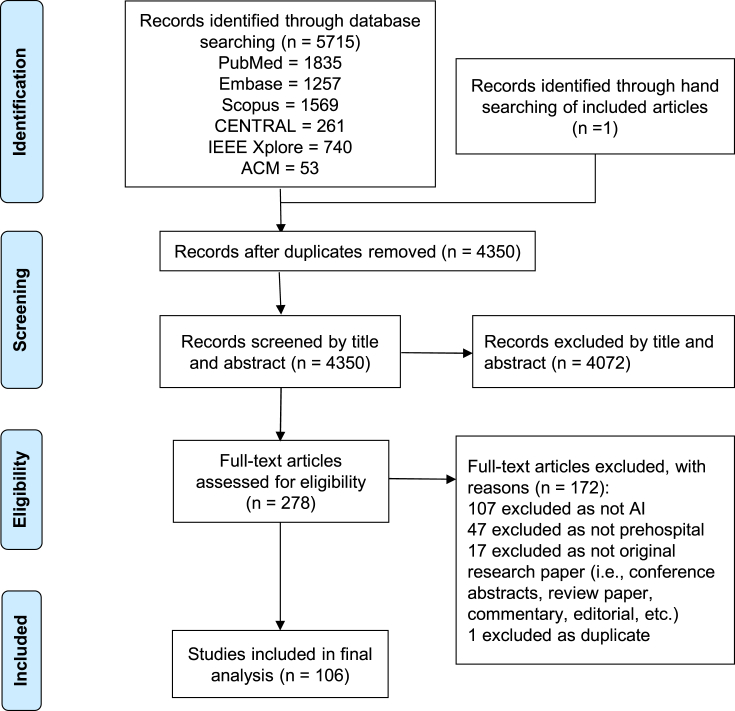
PRISMA flowchart of study selection.

Figure 1 shows the PRISMA flowchart of paper selection. The initial search of the six databases returned 4,349 papers and we identified one additional paper through hand searching of included articles. After excluding 4,072 papers on title and abstract screening, we identified 278 studies for full-text screening, of which 106 studies were included for data extraction and subsequent analysis.^15^^,^^16^^,^^17^^,^^18^^,^^19^^,^^20^^,^^21^^,^^22^^,^^23^^,^^24^^,^^25^^,^^26^^,^^27^^,^^28^^,^^29^^,^^30^^,^^31^^,^^32^^,^^33^^,^^34^^,^^35^^,^^36^^,^^37^^,^^38^^,^^39^^,^^40^^,^^41^^,^^42^^,^^43^^,^^44^^,^^45^^,^^46^^,^^47^^,^^48^^,^^49^^,^^50^^,^^51^^,^^52^^,^^53^^,^^54^^,^^55^^,^^56^^,^^57^^,^^58^^,^^59^^,^^60^^,^^61^^,^^62^^,^^63^^,^^64^^,^^65^^,^^66^^,^^67^^,^^68^^,^^69^^,^^70^^,^^71^^,^^72^^,^^73^^,^^74^^,^^75^^,^^76^^,^^77^^,^^78^^,^^79^^,^^80^^,^^81^^,^^82^^,^^83^^,^^84^^,^^85^^,^^86^^,^^87^^,^^88^^,^^89^^,^^90^^,^^91^^,^^92^^,^^93^^,^^94^^,^^95^^,^^96^^,^^97^^,^^98^^,^^99^^,^^100^^,^^101^^,^^102^^,^^103^^,^^104^^,^^105^^,^^106^^,^^107^^,^^108^^,^^109^^,^^110^^,^^111^^,^^112^^,^^113^^,^^114^^,^^115^^,^^116^^,^^117^^,^^118^^,^^119^^,^^120^

### Study characteristics and methodology

Table 1 shows the characteristics and methodology of the included studies (for raw data used for Table 1, see Table S4). Datasets from included studies were collected from 25 different countries. Most studies utilized datasets from North America or Europe, with data from the US being the most common (n = 46, 32.4%), followed by Sweden (n = 12, 11.3%), Norway (n = 11, 10.4%), Japan (n = 9, 8.5%), and the UK (n = 8, 7.5%).

**Table 1:** Study characteristics and methodology

Variable	n (%)
**AI type**	

Classification tree	2 (1.9)
Decision tree	6 (5.7)
Gradient boosted algorithm	2 (1.9)
Linear classifier	1 (0.9)
Mixed models	40 (37.7)
NLP	1 (0.9)
Neural network	28 (26.4)
Support vector machine	6 (5.7)
Random forest	14 (13.2)
Combined	10 (9.4)
Comparison	30 (28.3)
Other	2 (1.9)

**Comparator**	

Clinical decision tools	10 (9.4)
Human comparator	5 (4.7)
Other AI	22 (20.8)
Non-AI statistical models	10 (9.4)
Other	2 (1.9)
None	57 (53.8)

**Study type**	

Development	4 (3.8)
Development + validation	93 (87.7)
Predictor finding	4 (3.8)
Validation only	5 (4.7)

**Study design**	

Retrospective cohort	88 (83.0)
Prospective cohort	17 (16.0)
Randomized controlled trial	1 (0.9)

**Calibration**	

Not reported	96 (90.6)
Reported	10 (9.4)

**Country (of dataset origin)**	

USA	46 (43.4)
Sweden	12 (11.3)
Netherlands	3 (2.8)
Austria	4 (3.8)
France	5 (4.7)
Norway	11 (10.4)
UK	8 (7.5)
Denmark	2 (1.9)
Canada	1 (0.9)
China	4 (3.8)
Czech Republic	2 (1.9)
Finland	2 (1.9)
Japan	9 (8.5)
Greece	2 (1.9)
Italy	3 (2.8)
Ireland	1 (0.9)
Korea	2 (1.9)
Hong Kong	1 (0.9)
Germany	1 (0.9)
Mexico	1 (0.9)
South Africa	3 (2.8)
Taiwan	3 (2.8)
Spain	2 (1.9)
Slovenia	1 (0.9)
Singapore	1 (0.9)

**Inputs**	

ECG	40 (37.7)
Audio	5 (4.7)
EHR	53 (50.0)
Text	8 (7.5)
Public	5 (4.7)
Temporal	5 (4.7)
GIS	8 (7.5)
Image	1 (0.9)
Video	0 (0.0)
Vitals	15 (14.2)
Multiple	37 (34.9)
Other	7 (6.6)

The majority of included studies utilized a retrospective cohort (n = 88, 83.0%), with a few prospective cohorts (n = 17, 16.0%). Only one (0.9%) study was evaluated using a randomized controlled trial.

**Figure 2:**
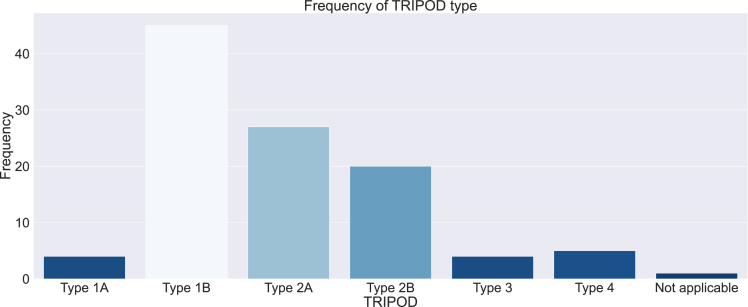
Frequency of TRIPOD types Type 1A, no validation, only evaluation of apparent model performance on the same training dataset; Type 1B, validation using re-sampling techniques such as bootstrapping or k-fold cross-validation; Type 2A, validation using a random split of data such as a train-test split; Type 2B, validation using a non-random split of data by time and/or location); Type 3, development and validation using separate sets of data; Type 4, evaluation of an existing model on separate data. Not applicable if study did not develop a predictive model.

Figure 2 shows the frequency of each TRIPOD type, with explanations of each type. Most studies were internally validated (n = 96, 90.6%). The most common TRIPOD classification was 1B (n = 45, 42.5%), where validation was done using re-sampling techniques. Type 2A (n = 27, 25.5%) and 2B (n = 20, 18.9%) were the next most common. Only 3.8% of studies (n = 4) were type 1A and did not perform validation. External validation is more robust but only 8.5% (n = 9) of studies used it; 3.8% (n = 4) were type 3, models were developed and validated on separate data, and 4.7% (n = 5) were type 4, where existing models were evaluated on separate data. One study^71^ was not classifiable as it was a predictor finding study that did not create a predictive model. Calibration was evaluated in only 9.4% (n = 10) of studies.

Included studies used a variety of AI types, with 37.7% of studies (n = 40) using multiple models. Of these, 10 studies (9.3%) combined models and 30 (28.3%) developed and compared multiple models. Among studies that developed a single AI model, 28 (26.4%) used neural networks, 14 (14.2%) used random forest, six (5.7%) used decision trees, six (5.7%) used support vector machines, two (1.9%) used classification trees, two (1.9%) used gradient boosted algorithms, one (0.9%) utilized a linear classifier, and one (0.9%) employed natural language processing.

### Longitudinal publication trends

**Figure 3:**
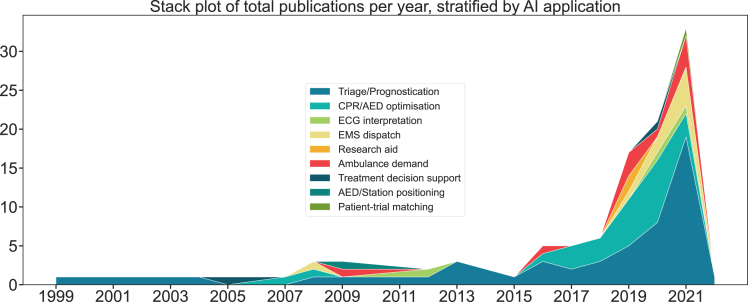
Stack plot of total publications by year, stratified by AI application.

Figure 3 shows the number of studies published per year, stratified according to AI application. Triage/Prognostication (n = 52, 49.1%) represented the majority of applications from 2015 onwards, with 57.6% in 2021. CPR/AED optimization publications (n = 26, 24.5%) also increased significantly from 2016, with 38.1% in 2020. The number of publications on AI in PEC increased sharply in 2019, peaking at 33 in 2021, compared to one in 2015. From 2017 to 2021, the diversity of AI applications also increased from two to six out of nine application types. Notably, remote monitoring (n = 2, 1.9%), research aid (n = 1, 0.9%), AED/station positioning (n = 1, 0.9%) and treatment decision support (n = 2, 1.9%) were underrepresented in the included studies.

### Performance of AI models against comparators

**Figure 4:**
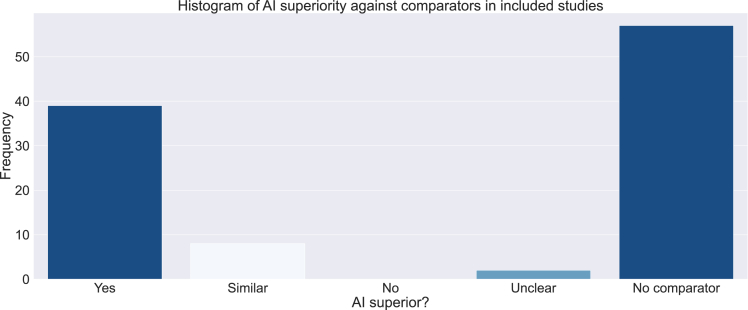
Histogram of AI superiority against comparators in included studies.

Figure 4 shows the performance of AI models against comparators in included studies. In this review, a comparator was defined as any benchmark of performance for the best-performing AI model in the study. AI and non-AI models developed as part of the same study were excluded as comparators. Fifty-seven (53.8%) studies did not use a comparator, 22 (20.8%) used other previously developed AI models, 10 (9.4%) used existing clinical decision tools, ten (9.4%) used non-AI statistical models, and five (4.7%) used human comparators. Two studies (1.9%) used comparators not included in these categories, such as baseline polices and baseline decision rules.^31^^,^^67^

Among 49 studies that used comparator against AI, AI was superior in 39 (79.6%) and not statistically different in 8 (16.3%). Results were unclear in two (4.1%) studies. No AI model performed worse than the comparator.

### Evidence map analysis

**Figure 5:**
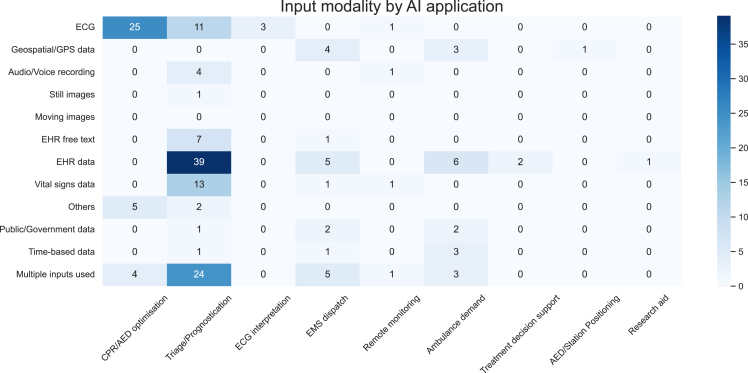
Evidence map of input modality by AI application See Data S1 section for more detailed explanations and examples of each input modality. Studies with multiple inputs are included in the count for each relevant input, as well as the count for multiple inputs. Hence, the sum of all cells is more than the number of included studies. ECG, electrocardiogram; EHR, Electronic Health Record; GPS, Global Positioning System.

We performed evidence map analysis to visualize the landscape of prehospital AI research and identify gaps, as demonstrated in previous reviews for AI in COVID-19 research.^13^ Figure 5 shows the evidence map of input modality compared against application type. CPR/AED optimization heavily relies on ECG (electrocardiogram) (25 out of 26) as an input and tends to be single input (22 out of 26). Triage/Prognostication tended to have multiple inputs (24 out of 52), with the majority (39 out of 52) using Electronic Health Records (EHR). Inputs such as ECG (40 out of 106), EHR (53 out of 106) and vitals (15 out of 106) were among the most used. The minority of studies utilized multimodal inputs (n = 37, 34.9%), and few models used text (n = 8), audio (n = 5), images (n = 1), or videos (n = 0) as inputs. Seven studies used inputs that did not fall into one of our predefined categories; these inputs included capnography,^38^^,^^107^ thoracic impedance,^19^^,^^38^^,^^100^^,^^101^^,^^117^ and accelerometer-based chest compression depth data.^98^

## Discussion

Recently, interest in AI and its applications in PEC has been rapidly growing, with diverse applications promising improvements to PEC systems globally. In this scoping review, we present the first overview of AI applications in PEC settings, including an evidence map analysis of current implementation gaps. AI applications in PEC have been reported to be superior to clinicians or non-AI algorithms, particularly in predictive tasks. Applications of AI in PEC are also diverse, including triage, resource optimization for dispatch, and geospatial optimization for stations and AEDs. However, gaps remain in the utilization of multimodal inputs such as text, audio, images, and video. In this discussion, we summarize the main findings of our review and provide insight into the potential benefits and challenges of AI in prehospital care.

We found that, like other areas of medicine, the most prevalent application of AI in PEC is triage and prognostication, in the form of diagnostic and prognostic predictive models. These models excel as rapid, objective tools for triage and prognostication in PEC settings, where clinician decision making is often time-sensitive. Prognostic models help identify patients who may be at high risk for poor outcomes, allowing for earlier intervention and management. Works by Liu et al.^80^^,^^81^ demonstrate how the combination of different features such as vital signs and heart rate variability and complexity in an ML prognostic model can provide an accurate estimation of risk in the prehospital setting. These models based on neural networks and multilayer perceptrons can accurately assess the need for lifesaving interventions in trauma patients in real-time. The works of Liu et al. highlight the capability of AI to harness advances in technology and healthcare big data for real-time, continuous monitoring and processing of in-ambulance data, such as vital signs and ECG signals. Similarly, Czap et al.^33^ have taken advantage of developments in mobile stroke units (MSUs) and validated an AI algorithm for the prehospital identification of large vessel occlusion using MSU CT angiograms.

Another major domain in prehospital prognostication is out-of-hospital cardiac arrest (OHCA). AI algorithms have been employed in the prediction of defibrillation success, as well as short- and long-term outcomes following OHCA. Patient outcomes may be improved with further research on the utility of these models in influencing early intervention and other treatment decisions in certain high-risk patients after OHCA.

Moreover, AI has been extensively used to tackle various optimization problems within PEC settings. Several studies have demonstrated the feasibility of AI-assisted dispatch systems to significantly improve response times and increase the efficiency of EMS operations. These studies mainly employ AI for the prediction of travel time^21^^,^^27^^,^^31^^,^^36^^,^^92^^,^^115^ and ambulance demand,^29^^,^^48^^,^^49^^,^^57^^,^^69^^,^^78^^,^^79^^,^^85^^,^^99^^,^^106^ which can assist with the generation of spatial coverage plans for EMS stations.^36^ Similarly, Mackle et al.^83^ used a genetic algorithm to simulate and optimize aerial AED drone positioning for quick access to patients in OHCAs, potentially improving long-term outcomes and survival rates.

We found several emerging use cases of AI in PEC. First, Stemerman et al.^112^ used clinical notes derived from the EMS to train ML algorithms for patient trial matching, potentially reducing the workload of research nurses and expediting research processes by automating workflows. Also of note is the emerging use of wearable IoT devices. Majumder et al.^84^ introduced an application of AI in pre-hospital patients using a wearable IoT device which signals the user’s OHCA risk with an approximate accuracy of 95%. Chan et al.^28^ investigated contactless detection of cardiac arrest through the integration of AI models that perform real-time classification of agonal breathing into smart IoT devices. With wearable IoT devices becoming more common, model inputs such as ECGs, vital signs, and potentially EHR will also become more readily accessible. With these rich information sources, there is significant potential for applying advanced AI and ML techniques^121^^,^^122^ and new physiological measures^123^ for remote continuous monitoring. However, such IoT systems are nascent and require further validation in larger datasets and real-world contexts.

The reported performance of AI applications has been encouraging, with several predictive models achieving areas under the receiver operating characteristic curve (AUROC) greater than 0.9 in their intended discriminatory tasks. However, we caution that these statistics may be optimistic. Many studies were internally validated (TRIPOD type 1A, 1B and 2A) with few studies employing appropriate temporal or spatial data splitting (type 2B) or external validation on datasets from other studies (type 3). Reporting of performance metrics such as calibration was also poor. It is thus uncertain whether the superior discrimination metrics reported in AI studies will translate to efficacy in real-world clinical scenarios which are more dynamic and heterogeneous. Regardless of performance, these AI applications are often the first decision support tools of their kind, with no previous benchmarks or comparators available. These applications represent new opportunities for decision support in triage and prognostication, resource optimization, and monitoring that have not been possible without AI. Rigorous validation and improved reporting will help to optimize these applications for real-world implementation. We recommend that future authors consult AI-specific guidelines such as SPIRIT-AI, CONSORT-AI, and more recently, DECIDE-AI, to guide model development and reporting of results.^124^^,^^125^

AI has several advantages over traditional methods in PEC settings. It can effectively analyze and interpret high-dimensional data, including EHR data, images, and ECG signals.^18^^,^^24^^,^^45^ AI can also integrate multimodal data^126^ and model nonlinear relationships. Shandilya et al.^107^ demonstrate this with nonlinear feature extraction and fusion of multimodal capnographic and ECG signal data, achieving an AUROC of 93.8% in predicting defibrillation outcomes. Pirneskoski et al.^95^ and Spangler et al.^111^’s AI models for risk prediction of various short-term outcomes outperformed the National Early Warning Scores (NEWS) even when using the same variables, suggesting superior discrimination with nonlinear modeling. Performance was further improved when multimodal data were included.^95^ Several studies used NLP to analyze multimodal EHR free-text data and speech audio samples for OHCA identification^22^^,^^23^^,^^25^ or general triage,^42^ tasks which are challenging with traditional methods. Nonetheless, the inclusion of multimodal data do not always improve performance.^102^ Additional data modalities also introduce implementation challenges, such as privacy concerns and data acquisition.^126^ Currently, multimodal AI is feasible on a small scale, but these challenges and technical limitations hinder the integration of large and diverse datasets. PEC data is highly multimodal, including ECG signals, ultrasound^127^ and CT imaging,^128^ and image, video, and audio from body worn cameras^129^ or wearables.^130^^,^^131^ With progress in multimodal AI, we anticipate improved performance and greater diversity in PEC AI applications.

Despite clear advantages of AI in predictive performance and versatility, the lack of interpretability is a major barrier to implementation.^132^ Healthcare professionals are hesitant to accept predictions from AI models without rationale, particularly in high acuity PEC settings. Opaque AI models whose predictions cannot be easily understood, referred to as ‘black boxes’, raise ethical concerns as they can lead to biased decision making and lack of accountability for any adverse outcomes.^133^ Thus, researchers may instead opt to use interpretable non-AI methods, such as logistic regression, or less complex AI models.^134^ An example is Goto et al.’s^46^ work with simple, interpretable decision-trees for EMS triage. This solution often, but not always,^26^^,^^77^^,^^96^^,^^102^^,^^118^ results in poorer discrimination compared to more complex methods like neural networks and deep learning.^42^^,^^62^^,^^75^ The challenge, then, is appropriately applying AI or non-AI methods in consideration of the clinical context and acceptable limits for performance and interpretability.

A promising solution to model opacity is explainable AI, an approach that seeks to increase AI transparency while maintaining performance.^135^ Explainable AI techniques, such as feature attribution and model agnostic methods, can help practitioners understand the model’s decision-making process and identify potential biases. The shift toward explainable AI enables applications to evolve beyond mere black boxes and serve as valuable decision support tools for practitioners. Yet, at present, not all AI algorithms have suitable explainability methods. In such cases, Ghassemi et al.^136^ argue that rigorous validation processes can instill sufficient trust and minimize bias in AI models. While validation processes may serve as a stopgap measure, the field of explainable AI remains a critical area of research to ensure the continued progress and integration of AI in PEC settings.

AI in PEC is a growing field, with numerous promising applications such as prognostication, demand prediction, resource optimization, and IoT continuous monitoring systems. While the potential for AI in PEC is promising, it is crucial to be judicious in the application of AI in PEC settings and to avoid over-generalizing its capabilities. We recognize that the field of AI in PEC is still in its infancy and more prospective, externally validated studies are needed for AI to progress beyond the proof-of-concept stage to real-world clinical settings.

### Limitations of the study

Our study has several limitations. First, we excluded articles on military and disaster medicine, which some may consider relevant to PEC. Our search criteria were also limited to a pre-specified list of AI models which provided clarity to but may have excluded new forms of AI. Additionally, we only searched for peer-reviewed English language articles, which might have resulted in the omission of gray literature and non-peer-reviewed articles such as conference abstracts. These limitations may have resulted in underrepresentation of AI applications in non-English speaking countries. Indeed, the included studies were primarily conducted in Europe or North America, and studies such as those from China may have been missed. Given the scoping nature of the review, we also did not conduct a formal risk of bias analysis. However, despite these limitations, our review provides a systematic overview of the current literature on AI applications in PEC.

## STAR Methods

### Resource availability

#### Lead contact

Further requests for resources and materials should be directed to and will be fulfilled by the Lead Contact, Dr Nan Liu (liu.nan@duke-nus.edu.sg).

#### Materials availability

This study did not yield new unique reagents.

#### Data and code availability

• This paper analyses existing, publicly available data, they can be shared by the lead contact upon request.
• This paper does not report original code.
• Any additional information required to reanalyse the data reported in this paper is available from the lead contact upon request.

### Method details

We reported this scoping review according to the Preferred Reporting Items for Systematic Reviews and Meta-Analyses Extension for Scoping Reviews (PRISMA-ScR) checklist (see Table S1). A review protocol was developed but was not publicly registered.

#### Literature search and selection criteria

We performed a systematic literature search in six databases, namely, PubMed, Embase, Scopus, IEEE Xplore, ACM Digital Library, and CENTRAL from inception to 14 December 2021. We selected PubMed, Embase, and Scopus for their broad coverage of biomedical and general scientific literature, IEEE Xplore and ACM Digital library to capture more specialised research on AI, and CENTRAL for its focus on controlled trials. We combined two broad concept sets on AI and PEC to conduct our search. A truncated search strategy listing the first three keywords in each set is shown here: (“Artificial intelligence” OR “Deep learning” OR “Machine learning” OR …) AND (“emergency medical service” OR “emergency health service” OR “prehospital” OR …). See Table S2 for the full search strategy.

We included original, English language articles that applied AI to PEC data. In this review, we considered articles to have applied AI if they used any of the following AI models: random forest, support vector machine, K-nearest neighbours, neural networks (including deep learning), gradient boosted machine, classification and regression tree, clustering, or natural language processing. We defined PEC to include applications for cardiopulmonary resuscitation (CPR) and automated external defibrillators (AEDs), out-of-hospital cardiac arrests (OHCA), and ambulances or emergency medical service (EMS) stations, but excluded applications in disaster and military medicine. Articles were excluded if they were duplicated, abstracts, or reviews.

#### Literature selection and data extraction

We exported all extracted literature entries into Microsoft Excel (Office 365) for screening and selection. Each article was independently screened by title and abstract initially, and then full-text by two of three reviewers (MLC1, KM, KT). Discrepancies were resolved through discussions among the two reviewers until consensus was achieved. There was substantial inter-rater agreement, with 96.2% absolute agreement and Cohen’s kappa statistic=0.629 (see Table S3 for raw data). Subsequently, MLC1, MLC2, and HH conducted information extraction from the included literature and all authors reviewed the results. We retrieved information from full-text articles of all included studies, including publication year, study aims, country of dataset origin, AI methods used, comparators used and performance of AI against comparators, study design, sample size and outcomes of interests in predictive modelling studies, input types used, and a summary of each study. We also recorded the study type according to the transparent reporting of a multivariable prediction model for individual prognosis or diagnosis (TRIPOD) classification of predictive models.^137^ The TRIPOD classification describes whether a study conducted model development, model validation, or both, as well as the type of model validation, if applicable.

#### Evidence map analysis

To investigate the knowledge gap in the current literature, we conducted an evidence map analysis of selected studies. We categorized the studies into one of the following applications: “CPR/AED optimisation”, “Triage/Prognostication”, “ECG interpretation” (electrocardiogram interpretation), “EMS dispatch”, “Remote monitoring”, “Ambulance demand”, “Treatment decision support”, “AED/Station positioning”, and “Research aid” (e.g., patient-trial matching). For each study, we recorded if it used one or more of the following inputs: “ECG”, “Audio/Voice recording”, “EHR (electronic health record) data” (categorical or continuous data, e.g., patient age and sex, presence or absence of symptoms, laboratory tests), “EHR free text”, “Public/Government data” (including weather and population data), “Geospatial/GPS data” (e.g., GPS coordinates), “Time-based data” (e.g. season or month of the year), “Still images” (e.g. X-rays, photos), “Moving images” (e.g. videos of echocardiograms), “Vital signs data” (e.g., blood pressure, heart rate), “Others”. We also noted if multiple input types were used. We analysed application-input pairs by aggregating the total number of studies for each pair and identified any implementation gaps using the evidence map. Given the heterogenous nature of PEC data, we wanted to analyse the trends in multimodal input utilisation and how different inputs are being used in each unique AI application.

#### Role of the funding source

The funder of the study had no role in study design, data collection, data analysis, data interpretation, or writing of the report.

## Supplemental information

Document S1. Tables S1 and S2

Table S3. All articles extracted, with screening decisions, related to Figure 1 and Method details

Table S4. Information extracted from included articles, related to Table 1
