# Supplementary material for: Artificial intelligence and machine learning in prehospital emergency care: A scoping review
Source: iScience. 2023 Jul 17;26(8):107407. doi: 10.1016/j.isci.2023.107407 (PMC10440716; doi:10.1016/j.isci.2023.107407)
Supplement: Document S1. Tables S1 and S2 [file mmc1.pdf]

iScience, Volume *26*

Supplemental information

Artificial intelligence and machine learning in prehospital emergency care: A scoping review

Marcel Lucas Chee, Mark Leonard Chee, Haotian Huang, Katelyn Mazzochi, Kieran Taylor, Han Wang, Mengling Feng, Andrew Fu Wah Ho, Fahad Javaid Siddiqui, Marcus Eng Hock Ong, and Nan Liu

Table S1: Preferred Reporting Items for Systematic reviews and Meta-Analyses extension for Scoping Reviews (PRISMA-ScR) Checklist (related to Method details)

SECTION	ITEM	PRISMA-ScR CHECKLIST ITEM	REPORTED ON PAGE #
**TITLE**			
Title	1	Identify the report as a scoping review.	1
**ABSTRACT**			
Structured summary	2	Provide a structured summary that includes (as applicable): background, objectives, eligibility criteria, sources of evidence, charting methods, results, and conclusions that relate to the review questions and objectives.	2
**INTRODUCTION**			
Rationale	3	Describe the rationale for the review in the context of what is already known. Explain why the review questions/objectives lend themselves to a scoping review approach.	3,4
Objectives	4	Provide an explicit statement of the questions and objectives being addressed with reference to their key elements (e.g., population or participants, concepts, and context) or other relevant key elements used to conceptualize the review questions and/or objectives.	4
**METHODS**			
Protocol and registration	5	Indicate whether a review protocol exists; state if and where it can be accessed (e.g., a Web address); and if available, provide registration information, including the registration number.	5
Eligibility criteria	6	Specify characteristics of the sources of evidence used as eligibility criteria (e.g., years considered, language, and publication status), and provide a rationale.	5
Information sources*	7	Describe all information sources in the search (e.g., databases with dates of coverage and contact with authors to identify additional sources), as well as the date the most recent search was executed.	5
Search	8	Present the full electronic search strategy for at least 1 database, including any limits used, such that it could be repeated.	5, S2
Selection of sources of evidence†	9	State the process for selecting sources of evidence (i.e., screening and eligibility) included in the scoping review.	5
Data charting process‡	10	Describe the methods of charting data from the included sources of evidence (e.g., calibrated forms or forms that have been tested by the team before their use, and whether data charting was done independently or in duplicate) and any processes for obtaining and confirming data from investigators.	5
Data items	11	List and define all variables for which data were sought and any assumptions and simplifications made.	5,6
Critical appraisal of individual sources of evidence§	12	If done, provide a rationale for conducting a critical appraisal of included sources of evidence; describe the methods used and how this information was used in any data synthesis (if appropriate).	Not within scope/Not appliable

SECTION	ITEM	PRISMA-ScR CHECKLIST ITEM	REPORTED ON PAGE #
Synthesis of results	13	Describe the methods of handling and summarizing the data that were charted.	5,6
**RESULTS**			
Selection of sources of evidence	14	Give numbers of sources of evidence screened, assessed for eligibility, and included in the review, with reasons for exclusions at each stage, ideally using a flow diagram.	7
Characteristics of sources of evidence	15	For each source of evidence, present characteristics for which data were charted and provide the citations.	7-9, S3
Critical appraisal within sources of evidence	16	If done, present data on critical appraisal of included sources of evidence (see item 12).	Not within scope/Not appliable
Results of individual sources of evidence	17	For each included source of evidence, present the relevant data that were charted that relate to the review questions and objectives.	7-10, S3
Synthesis of results	18	Summarize and/or present the charting results as they relate to the review questions and objectives.	9,10
**DISCUSSION**			
Summary of evidence	19	Summarize the main results (including an overview of concepts, themes, and types of evidence available), link to the review questions and objectives, and consider the relevance to key groups.	11-13
Limitations	20	Discuss the limitations of the scoping review process.	13
Conclusions	21	Provide a general interpretation of the results with respect to the review questions and objectives, as well as potential implications and/or next steps.	13
**FUNDING**			
Funding	22	Describe sources of funding for the included sources of evidence, as well as sources of funding for the scoping review. Describe the role of the funders of the scoping review.	2

JBI = Joanna Briggs Institute; PRISMA-ScR = Preferred Reporting Items for Systematic reviews and Meta-Analyses extension for Scoping Reviews.

* Where *sources of evidence* (see second footnote) are compiled from, such as bibliographic databases, social media platforms, and Web sites.

† A more inclusive/heterogeneous term used to account for the different types of evidence or data sources (e.g., quantitative and/or qualitative research, expert opinion, and policy documents) that may be eligible in a scoping review as opposed to only studies. This is not to be confused with *information sources* (see first footnote).

‡ The frameworks by Arksey and O'Malley (6) and Levac and colleagues (7) and the JBI guidance (4, 5) refer to the process of data extraction in a scoping review as data charting.

§ The process of systematically examining research evidence to assess its validity, results, and relevance before using it to inform a decision. This term is used for items 12 and 19 instead of "risk of bias" (which is more applicable to systematic reviews of interventions) to include and acknowledge the various sources of evidence that may be used in a scoping review (e.g., quantitative and/or qualitative research, expert opinion, and policy document).

From: Tricco AC, Lillie E, Zarin W, O'Brien KK, Colquhoun H, Levac D, et al. PRISMA Extension for Scoping Reviews (PRISMAScR): Checklist and Explanation. Ann Intern Med. 2018;169:467–473. doi: 10.7326/M18-0850.

Table S2 – Full search strategy for each database (related to Method details)

**Database**	**Search strategy**
Scopus	(TITLE-ABS ("Artificial* intelligen*" OR "Deep learning" OR "Machine learning" OR {AI} OR {pattern recognition} OR "comput* simulation" OR "data science" OR "decision support" OR "fuzzy logic" OR "fuzzy system" OR "Natural language*" OR {NLP} OR "neural network*" OR "nearest neighb*" OR {kNN} OR "Decision tree*" OR "Bayes*" OR "Random forest*" OR {RF} OR "Dimensionality reduction" OR "Principal component" OR {PCA} OR "Support vector" OR {SVM} OR "gradient boost*" OR "AdaBoost" OR "LightGBM" OR "XGBoost" OR "CatBoost")) AND (TITLE-ABS ("emergency medical service*" OR "emergency health service*" OR {prehospital} OR {pre-hospital} OR "ambulance*" OR "paramedic*" OR "out-of-hospital*" OR "automat* external defibrillat*")) AND NOT (TITLE-ABS-KEY (animal AND NOT {human})) AND (LIMIT-TO (SRCTYPE , "j")) AND (LIMIT-TO (DOCTYPE , "ar")) AND (LIMIT-TO (LANGUAGE , "English"))
PubMed	("artificial* intelligen*"[Title/Abstract] OR "Deep learning"[Title/Abstract] OR "Machine learning"[Title/Abstract] OR "AI"[Title/Abstract] OR "pattern recognition"[Title/Abstract] OR "comput* simulation"[Title/Abstract] OR "data science"[Title/Abstract] OR "decision support*"[Title/Abstract] OR "neural network*"[Title/Abstract] OR "fuzzy logic"[Title/Abstract] OR "fuzzy system*"[Title/Abstract] OR "natural language*"[Title/Abstract] OR "NLP"[Title/Abstract] OR "nearest neighb*"[Title/Abstract] OR "kNN"[Title/Abstract] OR "decision tree*"[Title/Abstract] OR "bayes*"[Title/Abstract] OR "random forest*"[Title/Abstract] OR "RF"[Title/Abstract] OR "Dimensionality reduction"[Title/Abstract] OR "Principal component"[Title/Abstract] OR "PCA"[Title/Abstract] OR "Support vector"[Title/Abstract] OR "SVM"[Title/Abstract] OR "gradient boost*"[Title/Abstract] OR "GBM"[Title/Abstract] OR "AdaBoost"[Title/Abstract] OR "LightGBM"[Title/Abstract] OR "XGBoost"[Title/Abstract] OR "CatBoost"[Title/Abstract]) AND ("emergency medical service*"[Title/Abstract] OR "emergency health service*"[Title/Abstract] OR "prehospital"[Title/Abstract] OR "pre-hospital"[Title/Abstract] OR "ambulance*"[Title/Abstract] OR "paramedic*"[Title/Abstract] OR "out of hospital*"[Title/Abstract] OR "automated external defibrillat*"[Title/Abstract])
IEEE Xplore	("Full Text & Metadata": Artificial intelligence" OR "Full Text & Metadata": "Deep learning" OR "Full Text & Metadata": "Machine learning" OR "Full Text & Metadata": "AI" OR "Full Text & Metadata": "pattern recognition" OR “Full Text & Metadata”: "comput* simulation" OR “Full Text & Metadata”data science" OR “Full Text & Metadata”decision support" OR “Full Text & Metadata”Neural network" OR “Full Text & Metadata”fuzzy logic" OR “Full Text & Metadata”: "fuzzy system" OR “Full Text & Metadata”: "Natural language" OR “Full Text & Metadata”NLP" OR “Full Text & Metadata”: "nearest neighb*" OR “Full Text & Metadata”: "kNN" OR “Full Text & Metadata”Decision tree" OR “Full Text & Metadata”: "Bayes*" OR “Full Text & Metadata”: "Random forest" OR “Full Text & Metadata”RF" OR “Full Text & Metadata”Dimensionality reduction" OR “Full Text & Metadata”: "Principal component" OR “Full Text & Metadata”: "PCA" OR “Full Text & Metadata”: "Support vector" OR “Full Text & Metadata”: "SVM" OR “Full Text & Metadata”: "gradient boost*" OR “Full Text & Metadata”: "GBM" OR “Full Text & Metadata”: "AdaBoost" OR “Full Text & Metadata”: "LightGBM" OR “Full Text & Metadata”: "XGBoost" OR “Full Text & Metadata”: "CatBoost") AND (“Full Text & Metadata”emergency medical service" OR “Full Text & Metadata”emergency health service" OR “Full Text & Metadata”prehospital" OR “Full Text & Metadata”pre-hospital" OR “Full Text & Metadata”ambulance" OR “Full Text & Metadata”paramedic*" OR “Full Text & Metadata”out-of-hospital" OR “Full Text & Metadata”automated external defibrillator" OR “Full Text & Metadata”automated external defibrillation")<br><br>+ limited to Journals only
Embase	Embase Classic+Embase <1947 to 2021 December 03>

	1. ("artificial* intelligen*" or "deep learning" or "machine learning" or "AI" or "pattern recognition" or " comput* simulation" or "data science" or "decision support* " or "neural network*" or "fuzzy logic" or "fuzzy system" or "natural language" or " NLP" or "nearest neighb*" or "kNN" or "Decision tree*" or "Bayes*" or "Random forest*" or "RF" or "Dimensionality reduction" or "Principal component" or "PCA" or "support vector" or "SVM" or "gradient boost*" or "GBM" or "AdaBoost" or "LightGBM" or "XGBoost" or "CatBoost").ab,ti. 545091<br>2. ("emergency medical service*" or "emergency health service*" or "prehospital" or "pre-hospital" or "ambulance*" or "paramedic*" or "out-of-hospital*" or "automated external defibrillat*").ab,ti. 72110<br>3. 1 and 2 892
ACM Digital Library	(Title:("Artificial* intelligen*" OR "Deep learning" OR "Machine learning" OR "AI" OR "pattern recognition" OR "comput* simulation" OR "data science" OR "decision support*" OR "Neural network*" OR "Natural language*" OR "NLP" OR "nearest neighb*" OR "kNN" OR "Decision tree*" OR "Bayes*" OR "Random forest*" OR "RF" OR "Dimensionality reduction" OR "Principal component" OR "PCA" OR "Support vector" OR "SVM" OR "gradient boost*" OR "GBM" OR "AdaBoost" OR "LightGBM" OR "XGBoost" OR "CatBoost") OR Abstract:("Artificial* intelligen*" OR "Deep learning" OR "Machine learning" OR "AI" OR "pattern recognition" OR "comput* simulation" OR "data science" OR "decision support*" OR "Neural network*" OR "Natural language*" OR "NLP" OR "nearest neighb*" OR "kNN" OR "Decision tree*" OR "Bayes*" OR "Random forest*" OR "RF" OR "Dimensionality reduction" OR "Principal component" OR "PCA" OR "Support vector" OR "SVM" OR "gradient boost*" OR "GBM" OR "AdaBoost" OR "LightGBM" OR "XGBoost" OR "CatBoost")) AND (Title:("emergency medical service*" OR "emergency health service*" OR "prehospital" OR "pre-hospital" OR "ambulance*" OR "paramedic*" OR "out-of-hospital*" OR "automated external defibrillat*") OR Abstract:("emergency medical service*" OR "prehospital" OR "pre-hospital" OR "ambulance*" OR "paramedic*" OR "out-of-hospital*" OR "automated external defibrillat*"))
Cochrane CENTRAL	1. ("Artificial* intelligen*" OR "Deep learning" OR "Machine learning" OR "AI" OR "pattern recognition" OR "comput* simulation" OR "data science" OR "decision support*" OR "Neural network*" OR "fuzzy logic" OR "fuzzy system" OR "Natural language*" OR "NLP" OR "nearest neighb*" OR "kNN" OR "Decision tree*" OR "Bayes*" OR "Random forest*" OR "RF" OR "Dimensionality reduction" OR "Principal component" OR "PCA" OR "Support vector" OR "SVM" OR "gradient boost*" OR "GBM" OR "AdaBoost" OR "LightGBM" OR "XGBoost" OR "CatBoost"):ti OR ("Artificial* intelligen*" OR "Deep learning" OR "Machine learning" OR "AI" OR "pattern recognition" OR "comput* simulation" OR "data science" OR "decision support*" OR "Neural network*" OR "fuzzy logic" OR "fuzzy system" OR "Natural language*" OR "NLP" OR "nearest neighb*" OR "kNN" OR "Decision tree*" OR "Bayes*" OR "Random forest*" OR "RF" OR "Dimensionality reduction" OR "Principal component" OR "PCA" OR "Support vector" OR "SVM" OR "gradient boost*" OR "GBM" OR "AdaBoost" OR "LightGBM" OR "XGBoost" OR "CatBoost"):ab<br>2. ("emergency medical service*" OR "emergency health service*" OR "prehospital" OR "pre-hospital" OR "ambulance*" OR "paramedic*" OR "out-of-hospital*" OR "automated external defibrillat*"):ti OR ("emergency medical service*" OR "emergency health service*" OR "prehospital" OR "pre-hospital" OR "ambulance*" OR "paramedic*" OR "out-of-hospital*" OR "automated external defibrillat*"):ab<br>3. #1 AND #2 (261)
